# King's College Hospital

**Published:** 1889-12-21

**Authors:** 


### The Hospital Workshop.

### No. XVIII.—KING'S COLLEGE HOSPITAL.

(By our Special Commissioner).

Between old Temple Bar and Holborn used to be some of the worst slums and rookeries in London. A few of these dens still exist on the Drury-lane side of the locality mentioned, but the greater part have been swept away to make room for a number of buildings more or less palatial. At the present day, if the reader take a map, he will find stretching north from the Griffin that marks the site of Temple Bar, first, the New Law Courts, then King's College Hospital, the College of Surgeons, Lincoln's-inn-fields, and, lastly, the huge block of the Inns of Court Hotel, extending from the Fields to its main frontage in Holborn.

King's College Hospital was founded fifty years ago, on the site of the old workhouse of St. Clement Danes, and had accommodation for 120 beds, as against an average of 162 occupied daily during 1888. The new building, erected in 1861, is just one of those solid and unpretentious-looking places that people learn to like upon acquaintance. On the front page of the committee's report is a lithographed picture of the hospital, presented by the *Graphic*. From a notice over the drawing—which is quite a work of art in its way—we see that this institution is "free and unendowed." Admission is given to the sick without subscribers' letters, although at the same time that system i followed to a certain limited extent. For instance, taking at random a couple of sheets from the in-patient register it was seen that ten out of each fifty were admitted upon recommendation. Provision is made for paying patients at one guinea per week, but it appears to be little used, since payments from that source during 1888 amount to £7 11s. only. The hospital may, therefore, claim to be practically "free," but the assertion that it is "unendowed" requires some further explanation in face of the fact that the balance-sheet for 1888 shows some £627 under the heading of receipts from dividends and rents. As to enquiry into the means of applicants for relief it is stated that although no special officer is appointed for that purpose, yet rejections are sometimes made. The occupation is entered in each case, and a special column is reserved for a note of amount of wages earned, but that does not appear to have been filled up for some years past. Information on this and other points was courteously afforded your commissioner by the Rev. N. Bromley, warden of the hospital.

The board-room is a handsome apartment with a ceiling in the delicate style that one associates with Wedgewood china and Chippendale furniture. On the mantelpiece is a striking portrait of one of the founders, and adjoining it is a duplicate of the picture by Mr. J. Yates Carrington, representing a wounded dog brought to the hospital by two friendly companions, a now famous bit of imaginative sentiment. Going along the central corridor and past the front entrance one walks into an extremely fine hall, containing the main staircase. It is square, lofty, lighted with many windows back and front, and covered in with a ceiling broken into massive flat alcoves. Running along the sides and opening on to the wards are three galleries with connecting staircases. The floor is composed of large lozenge-shaped slabs of stone, and its ample space is relieved by a single marble figure. Fixed against the wall underneath the first flight of steps is an interesting little memorial with the following history. This end of the building stands on what was formerly one of the old City graveyards. When this wing was added upon the site of the burial ground the human remains were carefully removed along with the gravestones. One of the latter proved of such interest that its inscription was carefully copied upon a memorial-stone, which was duly set up in the position mentioned. It is none other than the tombstone of the famous Joe Miller, the humourist, whose name has become a household word. The date of his death was 1738, and of the removal of his remains 1816.

This hospital is so arranged that each member of the medical staff has his own wards, a plan that certainly presents advantages over the more common one, which scatters patients over different parts of the building. A small detail worth notice is that the name of each ward is put up in good bold letters near the entrance. Under the guidance of Mr. P. T. Beale, house surgeon, your commissioner visited a male surgical ward under the care of Mr. Watson Cheyne, a well-known authority in the modern science of bacteriology. King's may be looked upon as the headquarters of antiseptic surgery, for its founder, Sir Joseph Lister, is one of the surgeons to the hospital. This system has simply revolutionised the whole practice of modern surgery, and has drawn into its net a large number of cases that have hitherto belonged to the physician. In this way diseases of the liver, of the kidney, of the brain, and of other internal organs, now come under the care of the operating surgeon.

The wards are of a homely, comfortable type, each being provided with a large handsome fireplace. The floors are of unpolished deal, and the walls distempered in various warm-tinted colours. Each ward contains fifteen beds on an average, and the floor-space is unusually wide, giving an ample allowance of air to each patient. Ventilation is effected chiefly by the windows, the top panes of which open by a slant, and the lower ones in the ordinary manner. Other sanitary arrangements appear to be good, and a feature worth notice is the method of disposal of all infected or foul linen, which is carried off by a special shoot to some underground region, where it is at once plunged into a bath of one to twenty carbolic acid solution.

A man is sitting by the fire with his head wrapped up in sky-blue bandages. The colour is not, as might be imagined at first sight, assumed for the sake of mere appearances, but has a far deeper and more scientific purpose. The drug known as salalembroth—a double salt of bichloride of mercury and chloride of ammonium—has of late been extensively used as an antiseptic, and materials charged with it have been commonly stained with an aniline blue so as to mark them at a glance. Another important point is that if discharges soak through the dressings a discoloured patch will at once appear on the bandage and put the surgeon on his guard. A short while since Sir Joseph, after many years of patient research and experiment, publicly announced the discovery of a new antiseptic, a double cyanide of mercury and zinc in combination with starch. It is unirritating to the skin, and appears to be nearly perfect as an antiseptic, being fatal to germs themselves as well as preventing their development. The professor offers this material to believers in his theory as the best he has yet been able to discover.

The history of a case lately in these wards shows well what surgery can do nowadays for grave affections within the skull. The patient suffered in the first place from chronic disease of the bones of the ear, which led to extensive inflammation of the membrane lining the inside of the skull, known as the "dura mater." Abscess followed, and in order to thoroughly expose the parts it was found necessary to remove about six square inches of bone with the trephine. The dura mater was found to be dead, and had to be scraped away bodily. After the operation, the scalp, which had been lifted by the semi-lunar flap now generally adopted in these cases, was fastened back across the gap. Before the skull was opened the patient had grown gradually unconscious, a condition which continued, from first to last, about three weeks. He then began to show signs of returning consciousness, and from that time steadily recovered until he was able to leave the hospital, snatched, as it were, by antiseptic surgery from the very jaws of death. The gap of bone is now closing up; he is deaf in the ear first affected, as one would expect, but he can hear well enough on the other side.

Here is a boy of fourteen who has lost the right eye and had his nose completely destroyed by the ravages of a so-called "lupus." The disease has been arrested, and, by a plastic operation, a new nose has been formed out of a flap taken from the cheek. At present the new nose is somewhat squat and unshapely, but its appearance will much improve as time goes on. When the new nose was touched the boy at once referred the sensation to its proper place. At first the touch appears to the patient to be on the spot, wherever that may be, whence the flap has been taken. But, after a while, the transplanted portion takes to itself the sensations belonging to its new station. The real explanation of this phenomenon is obscure. Some say it is due to the formation of new nerves; others trace it to the joining of the fibrils of the two cut surfaces; and others, again, to a fresh education of nerve centres in the brain.

The quarters of the resident medical officers look com fortable and cosy. The operating theatre is a large and well-lighted room, provided with two most ingenious operating-tables of a pattern identical with those described in a former paper dealing with the Royal Free Hospital in Gray's-inn-road. They are the joint invention of the present matron of King's, Miss Monk, and one of the surgeons.

The whole of the top floor is devoted to the nurses, and the arrangements for their comfort and accommodation seem to be very perfect. For many years the nursing had been undertaken by the sisterhood of St. John's House, but since 1885 it has been taken entirely into the hands of the hospital management. Miss Monk has organised a private nursing staff, which is reported to be in a flourishing condition. Your commissioner was conducted round this branch of the hospital by Sister Sibbald, who gave him much interesting information that will form the basis of a future paper.

